# Use of Pentamidine As Secondary Prophylaxis to Prevent Visceral Leishmaniasis Relapse in HIV Infected Patients, the First Twelve Months of a Prospective Cohort Study

**DOI:** 10.1371/journal.pntd.0004087

**Published:** 2015-10-02

**Authors:** Ermias Diro, Koert Ritmeijer, Marleen Boelaert, Fabiana Alves, Rezika Mohammed, Charles Abongomera, Raffaella Ravinetto, Maaike De Crop, Helina Fikre, Cherinet Adera, Robert Colebunders, Harry van Loen, Joris Menten, Lutgarde Lynen, Asrat Hailu, Johan van Griensven

**Affiliations:** 1 University of Gondar, Gondar, Ethiopia; 2 Institute of Tropical Medicine, Antwerp, Belgium; 3 Médecins sans Frontières, Amsterdam, The Netherlands; 4 Drugs for Neglected Diseases Initiative, Geneva, Switzerland; 5 Médecins sans Frontières, Abdurafi, Ethiopia; 6 Department of Pharmaceutical and Pharmacological Sciences, KU Leuven, Leuven, Belgium; 7 School of Medicine, Addis Ababa University, Addis Ababa, Ethiopia; Institut Pasteur de Tunis, TUNISIA

## Abstract

**Background:**

Visceral leishmaniasis (VL) has become an important opportunistic infection in persons with HIV-infection in VL-endemic areas. The co-infection leads to profound immunosuppression and high rate of annual VL recurrence. This study assessed the effectiveness, safety and feasibility of monthly pentamidine infusions to prevent recurrence of VL in HIV co-infected patients.

**Methods:**

A single-arm, open-label trial was conducted at two leishmaniasis treatment centers in northwest Ethiopia. HIV-infected patients with a VL episode were included after parasitological cure. Monthly infusions of 4mg/kg pentamidine-isethionate diluted in normal-saline were started for 12months. All received antiretroviral therapy (ART). Time-to-relapse or death was the primary end point.

**Results:**

Seventy-four patients were included. The probability of relapse-free survival at 6months and at 12 months was 79% and 71% respectively. Renal failure, a possible drug-related serious adverse event, occurred in two patients with severe pneumonia. Forty-one patients completed the regimen taking at least 11 of the 12 doses. Main reasons to discontinue were: 15 relapsed, five died and seven became lost to follow-up. More patients failed among those with a CD4+cell count ≤ 50cells/μl, 5/7 (71.4%) than those with counts above 200 cells/μl, 2/12 (16.7%), (p = 0.005).

**Conclusion:**

Pentamidine secondary prophylaxis led to a 29% failure rate within one year, much lower than reported in historical controls (50%-100%). Patients with low CD4+cell counts are at increased risk of relapse despite effective initial VL treatment, ART and secondary prophylaxis. VL should be detected and treated early enough in patients with HIV infection before profound immune deficiency installs.

## Author Summary

Relapse of visceral leishmaniasis (VL) among HIV co-infected patients occurs universally. Evidence on the use of secondary prophylaxis especially in anthroponotic transmission regions was lacking. It was found out now that secondary prophylaxis in addition to antiretroviral therapy for VL in people with HIV infection is useful to decrease the relapse rate. However, this intervention is more effective when started before profound immune deficiency. Patients with low CD4 cell counts continued to relapse significantly despite the use of secondary prophylaxis as compared to those with high CD4 cell counts. Earlier VL case detection and management is crucial. This is the first adequately powered trial that has addressed the use of secondary prophylaxis for prevention of VL relapse in HIV co-infected patients.

## Introduction

Visceral leishmaniasis (VL) is a fatal-but treatable- disease caused by a protozoan belonging to the *Leishmania donovani* complex. While the Indian-subcontinent, East-Africa and Brazil share the major disease burden, it was long known as a rare pediatric disease in the Mediterranean basin. However, in the HIV-era, VL resurged in Southern Europe in adults with HIV co-infection [1] and has been a clinical challenge until highly-active antiretroviral therapy (ART) was introduced [2,3]. Today the co-infection is reported from 35 countries [4] and VL is an important opportunistic infection of HIV [5,6].

The profound immune deficiency in HIV/VL co-infection leads to poor treatment outcome and frequent recurrence of VL. A few case series studies showed 50% to 100% relapse in a year period without antileishmanial secondary prophylaxis [7–11] compared with less than 10% relapse in those without HIV [10]. Individuals with multiple episodes of VL described as active chronic VL were also reported [12].

Secondary prophylaxis for the prevention of VL relapse is recommended in international guidelines [13,14] based on a few case series and small sample size studies from Europe where VL is due to *L infantum* and transmission is zoonotic [8,9,11,15,16].

In northwest Ethiopia, where VL is caused by *L donovani* and transmission is anthroponotic, the HIV co-infection rate reaches 20 to 30% with up to 56% relapse in a year in patients on ART but without secondary prophylaxis [17]. Patients with low CD4+cell count and/or multiple relapse had an increased risk of (subsequent) relapse [17]. Using first line antileishmanial drugs (sodium stibogluconate, liposomal amphotericin B, paromomycin, miltefosine) as secondary prophylaxis risks for resistance development that can easily be transmitted in anthroponotic transmission regions [4]. Thus, we chose pentamidine, an aromatic diamidine that is not used in first intention because of toxicity but that was found to be safe when used as prophylaxis at a lower dose (3–4mg/kg every 3–4 weeks) than the daily (4mg/kg) therapeutic dosage [16,18,19]. The objective of the study was to assess the effectiveness, safety and feasibility of this intervention.

## Methods

### Ethics statement

The protocol of the study was approved by the Ethiopian Regulatory Authority (Food, Medicine, Health Care Administration and Control Authority, FMHACA), the National Research Ethics Review Committee (NRERC) and the Institutional Review Board of University of Gondar in Ethiopia. Additionally, it was also approved by the Ethics Review Board of Médecins Sans Frontières, and the Ethics Committee of Antwerp University Hospital, Belgium. All subjects were included in to the study after written informed consent was signed. Free treatment was provided. Patients were compensated for transport and food during their visits to the study sites. All study documents were kept confidential and were accessible for the study team, monitors and inspectors. Trained clinical trial monitors carried out two pre-study visits, one initiation visit and 6 monitoring visits according to the WHO and ICH Good Clinical Practices standards. Regulatory inspection was carried out by FMHACA at both sites during the study period. The independent Data and Safety Monitoring Board met five times during the study and assessed the progress of the study when every quarter of total sample recruitment was achieved. The protocol was registered in Clinicaltrials.gov (code NCT01360762).

### Study design

This was an open label, single arm trial designed to investigate the effectiveness, safety and feasibility of monthly pentamidine prophylaxis to prevent VL relapse in patients with HIV. The study has three phases, an initial 12 months of monthly pentamidine (main study period), six months extended treatment period (with monthly pentamidine) for those who remain with CD4 count less than 200cells/μl at the end of 12months follow-up, and a subsequent 12months follow-up after the prophylaxis to assess long term outcomes. The findings of the latter two phases will be published in the future.

### Study setting

The study was conducted in Northwest Ethiopia–at the Leishmaniasis Research and Treatment Center (LRTC) at University of Gondar Hospital (UoGH) and at the Abdurafi Health Centre. They are the largest leishmaniasis treatment centers in the region and are supported by the non-governmental organizations Drugs for Neglected Diseases initiative (DNDi) and Médecins sans Frontières respectively.

### Recruitment

Recruitment of the patients for the study proceeded in two steps. During pre-screening, age 18 or more years, parasitological diagnosis of VL, documented HIV test result and acceptable distance of residence from the trial centres for monthly follow-up were checked. Eligible patients were then approached for consent.

There were three types of study participants considered at increased risk of relapse. Patients presenting with active VL disease during the recruitment period were classified into two groups. Current primary cases were those presenting with VL disease for the first time and current relapse cases were patients presenting with two or more episode of VL. Those with active VL disease were admitted to the treatment centres for VL treatment and combination ART (initiated or continued). The drugs used to treat VL were sodium stibogluconate alone or in combination with paromomycin and Liposomal amphotericin B alone or in combination with miltefosine. Treatment of VL was prolonged or changed from one regimen to another when there was no cure with the initial regimen used. The current primary VL cases were included in the study after VL cure if they had a CD4+cell count less than 200 cells/μl or a WHO stage 4 HIV/AIDS defining condition (other than VL) while the current relapse cases were included in the study regardless of the CD4+cell and WHO stage of the co-morbidities. Patients who were treated for VL before the start of the study recruitment but in follow-up (taking ART) were defined as past VL cases and were included if their CD4+cell count at the time of screening for the study was less than 200 cells/μl or if they had a WHO stage IV-defining illness on presentation. All cases were included after ascertaining parasitological cure (no parasite on tissue aspirate microscopy).

Renal dysfunction (creatinine above twice the normal reference), diabetes, pregnancy and lactation, and chronic medical conditions (e.g. cardiac illnesses) were exclusion criteria.

### Intervention

Pentamidine isethionate (provided by Sanofi-Aventis) was started one month after VL cure for the current VL patients; and soon after the inclusion criteria were met for past VL cases. The monthly infusion was continued until the primary end points are met. Pentamidine isethionate 300mg powder was reconstituted with 5ml distilled water and the 4mg/kg (maximum 300mg) was drawn and further diluted in 200ml normal saline for infusion over one hour. Frequent blood pressure monitoring was done during infusion. Any adverse events observed were documented.

### Follow-up

At every visit weight, height, temperature, blood pressure, pulse, spleen and liver size and nutritional status were checked. Monthly full blood count, creatinine, liver function tests and blood glucose; every fourth month electrocardiography tracing and amylase level; and every sixth month CD4+cell count were monitored. HIV viral load determination was done only when clinically indicated and logistically possible. All adverse events were documented, and all the serious adverse events were reported to the sponsor and concerned Ethics Committee via a fast track procedure. Adherence to ART and co-trimesaxole was monitored by patient interview and pill count. The nationally recommended definition for ART adherence as “Good” missing less than three doses; “Fair” missing less than nine doses and “Poor” missing more than nine doses per month was used. Patients found with additional opportunistic infections and/or ART failure were managed according to the national Ethiopian guidelines [20]. During the monthly scheduled visits or the unscheduled visits of the patient the possibility of VL relapse was assessed (fever, weight loss, organomegaly, reduction in hematological profiles). Tissue aspiration and microscopy was done when VL relapse was suspected.

### Laboratory examinations

HIV was diagnosed followed the national diagnostic algorithm of using two sequential positive rapid test results; KHB (Shanghai Kehua Bio-engineering Co-Ltd, Shanghai, China) followed by STAT-PAK (Chembio HIV1/2, Medford, New York, USA). In case of discrepancy between the two tests, the Uni-Gold (Trinity Biotech PLC, Bray, Ireland) was used as a tie breaker in Gondar. In Abdurafi, the confirmation was done by ImmunoComb (Orgenics ImmunoComb II, HIV 1&2 Combfirm) after two positive rapid tests.

VL was diagnosed by tissue aspirates (spleen or bone marrow) and microscopy of the giemsa stains for *Leishmania* amastigotes. Tissue aspiration was repeated at the end of treatment to assess parasitological cure defined as no parasite on microscopy from the tissue aspirate. Splenic aspiration was avoided whenever the patients had bleeding tendency or the platelet count was less than 50,000/μl.

CD4^+^ T lymphocyte count was done at recruitment and every sixth months during follow-up using FACS counter (BD FACSCalibur flow cytometer, 2009, USA). The haematological analysis was done by a haematology analyser–Beckman Coulter A^c^T diff, Beckman Coulter Inc., 2003, USA.

### Endpoints

We report here the outcomes as assessed by the end of 12 months. The primary effectiveness outcome was time to relapse or death (all causes); while the primary safety outcome was the proportion of patients with pentamidine related serious adverse events (SAEs) or pentamidine related adverse events that led to the discontinuation of the drug. An adverse event was considered drug-related when the relationship was judged as possibly, probably or definitely related according to the treating physician. The primary outcome for feasibility was the proportion of patients that completed at least 90% of the scheduled visits (i.e., 11 out of 12 pentamidine administrations without experiencing relapse or drug-related SAEs).

Secondary variables of interest related to safety were ‘any drug related adverse event and ‘any SAE’; while feasibility-related variables included ‘the number of treatment discontinuations, interruptions, and additional clinical/therapeutic interventions needed’. Causes of death were analyzed as tertiary end points.

### Sampling method and sample size

Sample size was calculated with a required precision of 10% for primary effectiveness, 7.5% for main safety analysis and 12.5% for tolerability. The anticipated main analysis outcomes were a failure rate of 20% and a frequency of drug related SAEs of 10%. With these assumptions, the required sample size was 65. Allowing for 10% of patients lost to follow-up, the final sample size was calculated to be 72.

### Statistical methods

**Fig 1:**
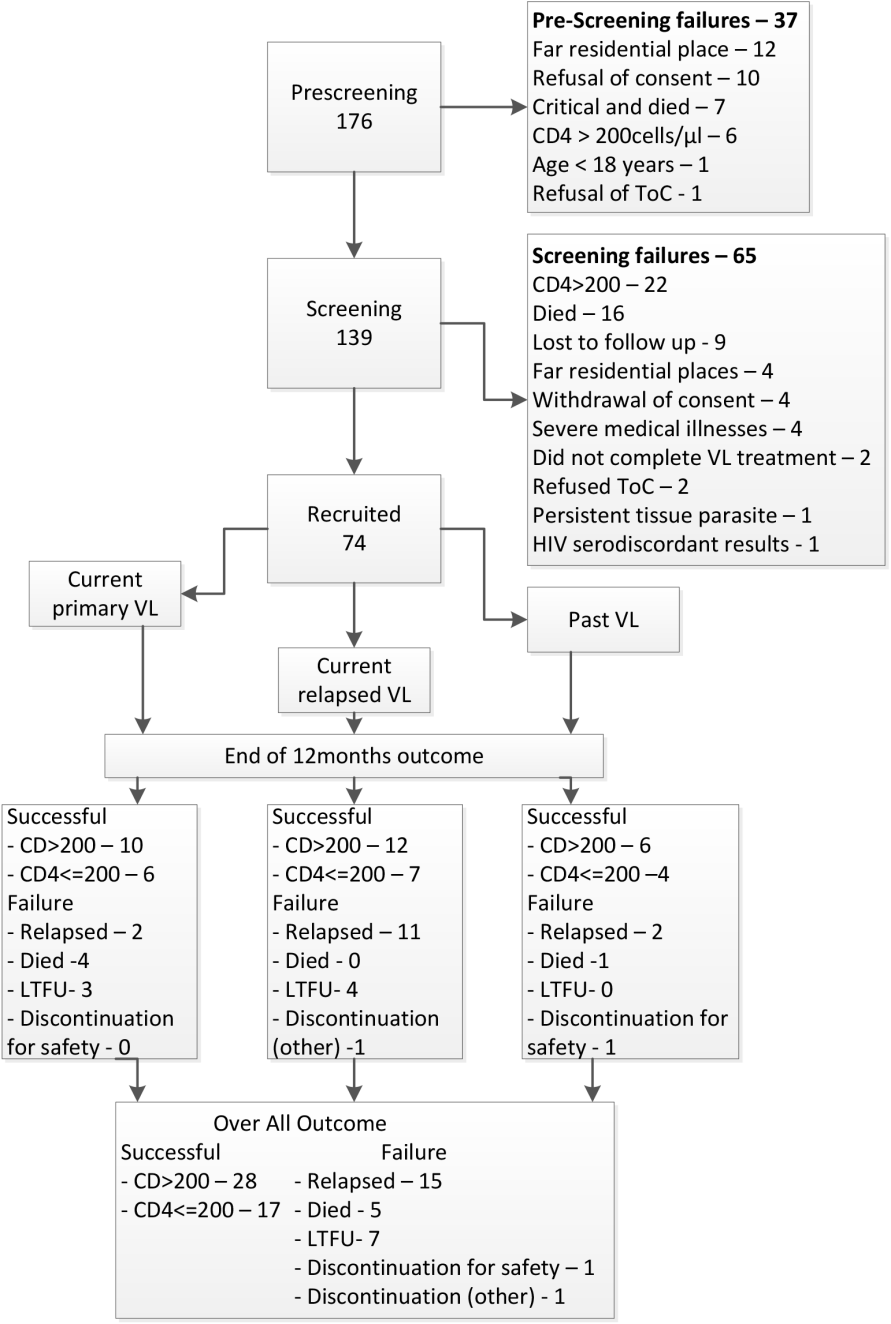
Flow chart showing the recruitment process and main outcomes.

A CONSORT diagram (Fig 1) and checklist (S1 Checklist) were used to present the patient accounting–total screened, screening failures, enrolled, discontinued and the outcomes. All patients who received a single dose of pentamidine were included in the analyses and the results presented for the three groups: primary VL, relapse and past VL. Baseline characteristics were presented in terms of medians and interquartile ranges for continuous characteristics and using counts and percentages for categorical characteristics.

Effectiveness: Effectiveness was analyzed using Kaplan-Meier survival analysis with time to relapse or death as outcome measure. "Failed" means the patient died or parasites were detected in tissues aspirates. Aspirates were taken in case of clinical suspicion of relapse. All other patients were considered free of relapse and were censored at 12 months (for patients who completed follow-up), or at their last visit for patients lost to follow-up. In principle, death was defined as all-cause mortality. The results were given as probability of relapse free survival with 95% confidence interval at 6 and 12 months. Patients who were lost to follow-up or discontinued the treatment for reasons not related to VL relapse before the end of the follow-up period were censored in the main study analysis, but were included as treatment failures in a “worst-case” scenario.

Safety: Adverse events were coded using the Medical Dictionary for Regulatory Activities (MEDDRA) and were analyzed based on counts of patients with a specific category and not on counts of individual AEs. Primary safety outcome was described in terms of counts of patients with drug-related SAEs or adverse events that led to study drug discontinuation. Counts (%) of patients with any SAE and any drug-related adverse events were presented as secondary safety outcomes.

Feasibility: The primary outcome for feasibility was the proportion of patients that completed at least 11 of the 12 monthly doses of the prophylaxis according to the protocol without experiencing relapse or drug-related SAEs expressed in percentage. The secondary feasibility endpoint was computed as the number (%) of patients who interrupted (temporarily or permanently), and the number of clinical interventions and/or therapeutic procedures needed.

## Results

### Study population

From a total of 176 HIV/VL patients, 74 were recruited into the study (38 at Abdurafi and 36 at Gondar) (Fig 1) in the period from November 2011 to September 2013. Most were male migrant workers, with a median age of 32 years. Sixty patients were current VL cases (25 primary and 35 relapsed VL cases), while the rest (14) were past VL cases. Demographics and baseline characteristics were similar among the three groups (Table 1).

**Table 1:** Baseline characteristics of recruited patients in three groups.

Characteristics	Current Primary	Current Relapse	Past	N (%)
Sex				
-Male	24 (96.0)	34 (97.1)	13 (92.9)	71 (96.0)
-Female	1 (4)	1 (2.9)	1 (7.1)	3 (4.1)
Age in years, median (IQR)	35 (28–39)	30 (27–35)	35 (30–42)	32 (28–37)
Site				
-Abdurafi	11 (44)	18 (51.4)	9 (64.3)	38 (51.4)
-Gondar	14 (56.0)	17 (48.6)	5 (35.7)	36 (48.7)
Body Mass Index (BMI)				
-BMI <18.5kg/m^2^	20 (80)	24 (68.6)	12 (85.7)	56 (75.7)
-BMI ≥ 18.5kg/m^2^	5 (20)	11 (31.4)	2 (14.3)	18 (24.3)
Spleen size: (n = 73)*				
-Not palpable: n (%)	7 (28)	16 (47.1)	7 (50)	30 (41.1)
-Palpable <5cm: n (%)	5 (20)	5 (14.7)	4 (28.6)	14 (19.2)
-Palpable ≥5cm: n (%)	13 (52)	13 (38.2)	3 (21.4)	29 (39.7)
Total liver span (cm): median (IQR)	11 (10–13)	12 (10.5–14)	10.5 (10–11)	11 (10–13)
Laboratory findings				
Total WBC count: median (IQR)	2600 (2300–3620)	3350 (2400–4185)	2920 (2100–3570)	3000 (2300–3900)
Neutrophil percent: median (IQR)	61.2 (48–70.4)	61.4 (51.9–70.7)	66.6 (50.0–69.3)	62.3 (48.4–70.6)
Lymphocyte percent: median (IQR)	29.7 (23.1–38.5)	27.3 (22.1–37.9)	27.6 (18.8–41.9)	27.8 (21.9–38.5)
Haemoglobin: median (IQR)	8.9 (7.1–10.8)	9.3 (8–10.7)	11.2 (8.8–13.2)	9.2 (7.7–11.1)
Platelet count (X1000): median (IQR)	197 (163–247)	214 (132.5–281.5)	153 (106–226)	192 (136–274)
CD4+cell count at recruitment: Median (IQR)	126 (95–157)	123 (91–219)	151.5 (79–185)	127 (91–185)
-≤ 50: n (%)	2 (8.7)	2 (6.1)	3 (21.4)	7 (10)
-51–100: n (%)	5 (21.7)	8 (24.2)	1 (7.1)	14 (20.0)
-101–200: n (%)	15 (65.2)	12 (36.4)	10 (71.4)	37 (52.9)
-201–350: n (%)	1 (4.4)	6 (18.2)	0	7 (10)
->350: n (%)	0	5 (15.2)	0	5 (7.1)
VL status				
-Primary	25 (100)	0	6 (42.9)	31 (41.9)
-Relapse	0	35 (100)	8 (57.1)	43 (58.1)
Frequency of relapse				
-1 relapse		22 (62.9)	5 (62.5)	27 (62.8)
-2 relapse		11 (31.4)	1 (12.5)	12 (27.9)
-3 relapse		2 (5.7)	1 (12.5)	3 (7.0)
-4 relapse		0	1 (12.5)	1 (2.3)

*Not measured due to ascites, IQR–Interquartile range, bpm–beats per minute, VL–visceral leishmaniasis, WBC–white blood cells

Most were malnourished, 56/74 (76%) (body-mass-index less than 18.5kg/m^2^) and with a history of VL relapse, 43 (58%). The median duration on ART was 7 months. Tenofovir, lamivudine and efavirenz combination was the common (74%) ART regimen used. While the median CD4+cell count at ART initiation was 70cells/μl, it was 127 cells/μl at inclusion into the study, with 60 (85.7%) having a CD4+cell count below 200cells/μl (Table 2).

**Table 2:** HIV and VL treatment history.

Characteristics	Current Primary	Current Relapse	Past VL	N (%)
Months HIV was diagnosed, median, IQR	3 (2–9)	15.5 (8–33)	30 (12–35)	12 (3–29.5)
Months on ART, median, IQR	2 (1–8)	8 (2–25)	15 (8–33)	7 (2–15)
Current ART regimen				
-TDF+3TC+EFV	20 (80.0)	26 (74.3)	9 (64.3)	55 (74.3)
-TDF+3TC+NVP	2 (8.0)	5 (14.3)	2 (14.3)	9 (12.2)
-AZT+3TC+EFV	2 (8.0)	0	1 (7.1)	3 (4.1)
-AZT+3TC+NVP	1 (4.0)	2 (5.7)	1 (7.1)	4 (5.4)
-D4T+3TC+NVP	0	1 (2.9)	1 (7.1)	2 (2.7)
-ABC+DDI+LPV/r	0	1 (2.9)	0	1 (1.4)
CD4+cells at ART initiation, median (IQR) (n = 61)	84 (46–126)	67 (40–129)	59 (24–108)	70 (44–125)
-CD4+cells< = 50	6 (28.6)	12 (37.5)	3 (37.5)	21 (34.4)
-CD4+cells: 51–100	7 (33.3)	9 (28.1)	3 (37.5)	19 (31.2)
-CD4+cells: >100	8 (38.1)	11 (34.4)	2 (25.0)	21 (34.4)
Months since last VL, median, (IQR)	2 (2–2)	2 (2–3)	4.5 (4–17)	2 (2–3)
Antileishmania drugs used during the last episode VL*				
-Sodium stibogluconate	12 (48.0)	18 (51.4)	8 (57.1)	38 (51.4)
-Liposomal amphotericin B	14 (56.0)	25 (71.4)	10 (71.4)	49 (66.2)
-Miltefosine	12 (48)	22 (62.9)	7 (50.0)	41 (55.4)
-Paromomycin	4 (16.0)	5 (14.3)	0	9 (12.2)
WHO Stage (excluding VL)				
-Stage 1	10 (40.0)	18 (51.4)	4 (28.6)	32 (43.2)
-Stage 2	7 (28.0)	4 (11.4)	4 (28.6)	15 (20.3)
-Stage 3	4 (16.0)	5 (14.3)	2 (14.3)	11 (14.9)
-Stage 4	4 (16.0)	8 (22.9)	4 (28.6)	16 (21.6)
Antituberculosis treatment	2 (8.0)	4 (11.4)	0	6 (8.1)

VL–visceral leishmaniasis, ART–antiretroviral therapy, IQR–interquartile range, TDF–tenofovir, 3TC–lamivudine, EFV–efavirenze, AZT–zidovudine, NVP–nevirapine, D4T –didanosine, DDI–didanosine, ABC–abacavir, LPV/r–lopinavir/ritonavir

*Miltefosine and paromomycin were always used in combination with Liposomal amphotericin B and sodium stibogluconate respectively. Prolonged treatments with different regimens were also done until cure was achieved.

### Outcomes

#### Effectiveness

**Fig 2:**
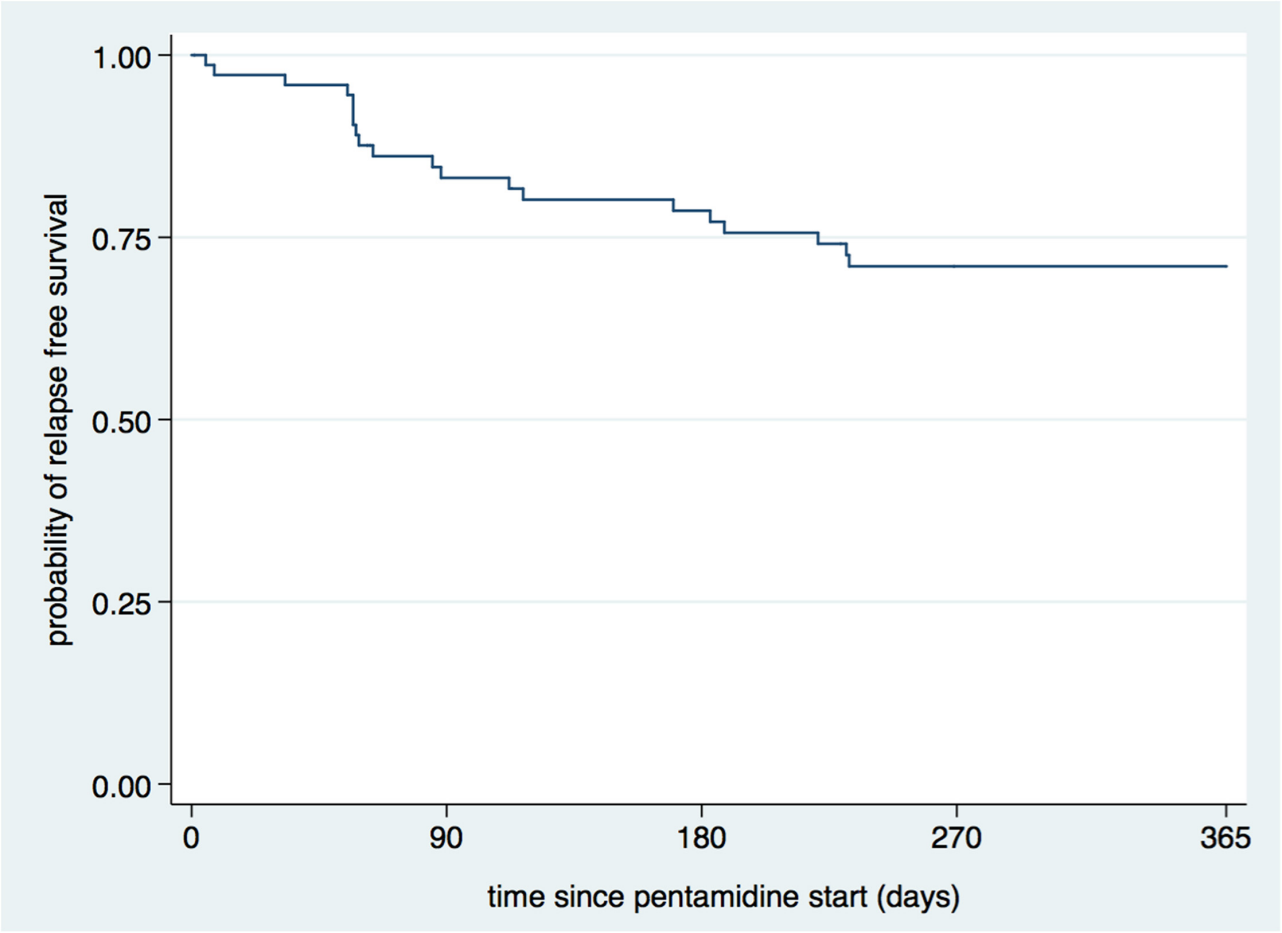
Kaplan-Meier survival estimate of the main effectiveness analysis.

The Kaplan-Meier estimated probability of relapse free survival at the end of 6 and 12 months was 79% (95% CI: 67%–87%) and 71% (95% CI: 59–80) respectively (Fig 2), and it was comparable among all the three patient groups. Counting all those who were lost to follow-up and who discontinued for safety or other reasons as failures (i.e. a worst-case scenario), the probability of relapse free-survival was 70% (95%CI: 58%–79%) at 6 months and 61% (95%CI: 49%–71%) at 12 months (Table 3).

**Table 3:** Summary of Primary Effectiveness Analysis Results.

	Month 6		Month 12	
	n failed / n censored	Probability Relapse Free** (95% CI)	n failed/ n censored	Probability Relapse Free (95% CI)
Primary Analysis				
All Patients	15/74	0.79 (0.67–0.87)	20 / 74	0.71 (0.59–0.80)
Current: primary	5/25	0.79 (0.57–0.90)	6/25	0.75 (0.52–0.88)
Current: relapse	8/35	0.76 (0.58–0.87)	11/35	0.66 (0.48–0.80)
Past VL case	2/14	0.85 (0.52–0.96)	3/14	0.77 (0.45–0.92)
Sensitivity Analysis: "worst case"				
All Patients	22/74	0.70 (0.58–0.79)	29 / 74	0.61 (0.49–0.71)
Current: primary	7/25	0.72 (0.50–0.86)	9/25	0.64 (0.42–0.79)
Current: relapse	12/35	0.66 (0.48–0.79)	16/35	0.54 (0.37–0.69)
Past VL case	3/14	0.79 (0.47–0.93)	4/14	0.71 (0.41–0.88)

**Survival analysis percentages take into account that some patients were censored during the follow-up, VL–visceral leishmaniasis

#### Safety

Two patients hospitalized for severe pneumonia had renal failure that was possibly related to pentamidine. One patient had to stop the study drug due to hyperglycemia though it normalized within a month.

There were a total of 21 SAEs (excluding admissions due to VL relapse) that occurred in 17 (23%) patients (S1 Table); and 42 study-drug related adverse events (all forms) in 30 (40.5%) of the study participants during the 12 months follow-up. The most common drug-related adverse events were symptoms of the respiratory system (nasal congestion) during pentamidine infusion– 14 (19%), hypotension– 11 (15%) and renal impairment—5 (6.8%). Other adverse events possibly related to pentamidine were injection site reactions, hypoglycemia, ocular hyperemia, headache, arthralgia and tetany (Table 4). SAEs were mainly due to infections, 11 due to severe pneumonia; and meningitis, severe diarrhea, disseminated tuberculosis, bacterial lymphadenitis and herpes zoster each occurred once. Renal failure needing admission occurred in two and upper gastrointestinal bleeding, hypovolemic shock and hypoglycemia each also happened once. The list of all adverse events classified with organ system and in MEDRA term is available in S2 Table.

**Table 4:** Drug related adverse events.

Drug related adverse events (serious and non-serious)	Current: primary (N = 25)	Current: relapse (N = 35)	Past VL (N = 14)	All (N = 74)
Ocular hyperaemia	0	1 (3)	0	1 (1)
Application site hypersensitivity	1 (4)	3 (8.6)	0	4 (5.4)
Renal impairment	3 (12)	1 (3)	1 (7)	5 (6.8)*
Hyperglycemia	0	0	1 (7)	1 (1)**
Hypoglycaemia	1 (4)	1 (3)	0	2 (3)
Tetany	0	0	1 (7)	1 (1)
Arthralgia	0	1 (3)	0	1 (1)
Headache	0	2 (6)	0	2 (3)
Nasal congestion	6 (24)	8 (23)	0	14 (19)
Hypotension	3 (12)	4 (11)	4 (29)	11 (15)
Total	11 (44)	14 (40)	5 (36)	30 (41)

VL–visceral leishmaniasis

*two of the renal impairments occurred in patients admitted for severe pneumonia who eventually died while on treatment. But as pentamidine is also known for its renal effect, they were considered as possibly pentamidine related serious adverse events

**this patient was made to discontinue the pentamidine when he developed hyperglycemia that normalized on the next month visit. These three patients (2 with renal failures and 1 with hyperglycemia) accounted for the primary safety outcome)

#### Feasibility

Forty-one (55%) of the participants completed the follow-up taking at least 11 of the planned 12 doses without experiencing relapse, death or drug-related SAEs. Four patients who missed more than one dose still had a successful outcome. The remaining 29 patients discontinued pentamidine permanently; 15 (20.3%) of them because of relapse, 7 (9.5%) were lost to follow-up, 5 (6.8%) died, one patient had to stop due to hyperglycemia and another patient refused to take the study drug.

Clinical and therapeutic interventions for pentamidine related adverse events were needed for 11 (14.9%) of the study participants. The common therapeutic interventions were additional intravenous fluid during pentamidine administration and reducing the rate of pentamidine infusion each happening ten times. Oral hydrations, prolonged hospital observation and additional medication during pentamidine infusion were each required twice and once glucose supplementation was done.

#### Mortality

There were five deaths, three in hospital and two out of the hospital, mainly due to infections. Two patients with severe pneumonia and renal failure died in hospital. The other hospital death was due to meningitis. One patient died of cryptosporidial diarrhea. The cause of death of the fifth patient was considered to be severe pneumonia based on verbal description from his relatives.

### Risk factors for failure of secondary prophylaxis

There were 5/7 (71.4%) failures among patients with a CD4^+^cell count ≤ 50cells/μl, whereas 2/12 (16.7%) failed in those with a CD4^+^cell count greater than 200cells/μl (p = 0.005) (Table 5). Age, body mass index, presence of previous relapse or the number of VL episodes, the antileishmanial drug used to treat the most recent VL episode, duration of ART (less than or greater than 6 months), adherence to ART (S3 Table) and diagnosis of tuberculosis did not show statistical significance with chemoprophylaxis failure.

**Table 5:** Risk factors for relapse.

Risk factors	Failure (relapse + death) n/N (%)	P	Failure (Worst-Case Scenario) n/N (%)	P
Sex		0.746		0.959
-Female	1/3 (33.3)		1/3 (33.3)	
-Male	19/71 (26.8)		28/71 (39.4)	
Age Category		0.305		0.340
-< 35 years	10/42 (23.8)		15/42 (35.7)	
->35 years	10/32 (31.3)		14/32 (43.8)	
Body mass index (BMI)		0.757		0.492
-BMI < 18.5kg/m^2^	15/56 (26.8)		21/56 (37.5)	
-BMI ≥ 18.5kg/m^2^	5/18 (27.8)		8/18 (44.4)	
VL type		0.556		0.375
-Current	17/60 (28.3)		25/60 (41.7)	
-Past	3/14 (21.4)		4/14 (28.6)	
VL status		0.251		0.174
-Primary	6/31 (19.4)		9/31 (29.0)	
-Relapse	14/43 (32.6)		20/43 (46.5)	
VL classification		0.749		0.556
-Current: primary	6/25 (24.0)		9/25 (36.0)	
-Current: relapse	11/35 (31.4)		16/35 (45.7)	
-Past VL	3/14 (21.4)		4/14 (28.6)	
Relapse Category		0.164		0.307
-0	6/31 (19.4)		9/31 (29.0)	
-1	7/27 (25.9)		12/27 (44.4)	
-2	7/16 (43.8)		8/16 (50.0)	
Sodium stibogluconate use for last episode VL		0.778		0.968
-no	9/36 (25.0)		14/36 (38.9)	
-yes	11/38 (29.0)		15/38 (39.5)	
Liposomal amphotericin B use for last episode of VL		0.856		0.891
-no	7/25 (28.0)		10/25 (28.0)	
-yes	13/49 (26.5)		19/49 (38.8)	
Miltefosine use for last episode VL		0.953		0.980
-no	9/33 (27.3)		13/33 (27.3)	
-yes	11/41 (26.8)		16/41 (39.0)	
ART duration		0.099		0.103
-≤6months	6/35 (17.1)		10/35 (28.6)	
->6months	13/38 (34.2)		18/38 (47.4)	
Baseline CD4+cell count (n = 70)		0.005		0.044
-≤50	5/7 (71.4)		5/7 (71.4)	
-51–100	6/15 (40.0)		8/15 (53.3)	
-101–200	6/36 (16.7)		11/36 (30.6)	
->200	2/12 (16.7)		3/12 (25.0)	
Anti-tuberculosis treatment		0.780		0.677
-No	18/68 (26.5)		26/68 (38.2)	
-Yes	2/6 (33.3)		3/6 (50.0)	

VL–visceral leishmaniasis, ART–antiretroviral therapy

HIV viral load was done only for eight of the patients who failed (1 death and 7 relapse cases) and it was undetectable in five of them.

## Discussion

The probability of failure (relapse or death) from secondary VL chemoprophylaxis within 1 year was 29% which is lower than the 50% to 100% reported in case series without prophylaxis in Europe [7–11]. The annual probability of VL relapse was 56% in a cohort of patients with HIV on ART, but without secondary prophylaxis in northwest Ethiopia [17]. In a meta-analysis of studies conducted in the *L infantum* region, the relapse rate was reduced from 67% to 31% with chemoprophylaxis [21]. Our study endpoint was relapse and death, while only the relapse rate was reported from the other studies.

Our data corroborate the risk of relapse associated with low CD4^+^ cell counts, but not with previous multiple relapses as seen before in a study in Ethiopia [17]. However, this is a subgroup analysis and the sample size is not adequately powered to make conclusions. The majority (75%) of the failures occurred in the first six months of the follow-up. In east Africa, VL relapse usually occurs in the 3 to 9 months following the initial treatment [17]. Early relapse may actually be a treatment failure that was missed due to the inherently less sensitive microscopy leading to a false verdict parasitological cure. Or else it is related to a deficient cellular immunity to control remaining parasites after treatment, resulting in a regeneration of the parasite and another episode of disease. Additionally, the protective serum level of pentamidine might not be reached in the first few months. The optimal dose of pentamidine for prophylaxis is also not clearly known. While 4mg/kg dose was meant to be for base-moiety, guidelines did not specify the need for dose modification of the different salt preparations [22]. Similar to other studies, pentamidine prophylaxis was found to be safe [8,16,23–25]. Only one patient developed transient hyperglycemia. Although renal failure occurred in two patients ultimately leading to death, the patients were having severe infections and it was difficult to attribute the cause of renal failure solely to pentamidine. Other adverse events were mild.

Seven (9.5%) of the study participants were lost to follow-up and four (5%) of the patients interrupted more than one dose before the primary end point was met. Despite the fact that our study patients belonged to a highly mobile and difficult to trace population group (migrant workers), the proportion of lost to follow-up did not exceed the 10% accounted in the initial sample size calculation.

Our study has several limitations. It is not a randomized controlled trial because there was no other antileishmanial drug available or recommended by the Ethiopian national guidelines to compare with. Secondly, because international guidelines recommend secondary prophylaxis to prevent VL recurrence in HIV equipoise was hard to claim [13,14]. We did not systematically monitor HIV viral load and we did not determine pentamidine serum levels. Future research need to include pharmacokinetics and resistance testing for anti-leishmania drugs. The efficacy and safety of higher doses of pentamidine and/or more frequent dosing should also be explored.

In conclusion, longer VL relapse free survival was achieved using pentamidine as secondary prophylaxis in people with HIV infection. However, patients with profound immune deficiency were still at risk of relapse. Thus, there is a need to investigate additional treatment options for this group of patients. Early VL case detection (before profound immune deficiency) is crucial for effective management and prevention of relapses [26].

## Supporting Information

S1 Checklist CONSORT 2010 checklist of information. (DOC)

S1 Table The type and frequency of all serious adverse events during the 12 months of follow up. (DOCX)

S2 Table Adverse events in the 12 months follow up with use of MEDRA term and body system classification. (DOCX)

S3 Table Adherence to antiretroviral therapy and outcome (assessment done at each monthly visit of 12 months follow up). (DOCX)
