# Supplementary material for: Use of Pentamidine As Secondary Prophylaxis to Prevent Visceral Leishmaniasis Relapse in HIV Infected Patients, the First Twelve Months of a Prospective Cohort Study
Source: PLoS Negl Trop Dis. 2015 Oct 2;9(10):e0004087. doi: 10.1371/journal.pntd.0004087 (PMC4591988; doi:10.1371/journal.pntd.0004087)
Supplement: S1 Table — (DOCX) [file pntd.0004087.s002.docx]

**Supplemental table 1: The type and frequency of all serious adverse events during the 12 months of follow up**

| Any SAE | Current: primary  (N=25) | Current: relapse (N= 35) | Prior VL Cases (N=14) | All  (N=74) |
| --- | --- | --- | --- | --- |
| Diarrhoea | 0 | 0 | 1 (7) | 1 (1) |
| Upper gastrointestinal haemorrhage | 0 | 0 | 1 (7) | 1 (1) |
| Disseminated tuberculosis | 0 | 1 (3) | 0 | 1 (1) |
| Herpes zoster | 0 | 1(3) | 0 | 1(1) |
| Lympadenitis bacterial | 1(4) | 0 | 0 | 1(1) |
| Meningitis | 1 (4) | 0 | 0 | 1(1) |
| Pneumonia | 3 (12) | 4 (11) | 4 (29) | 11(15) |
| Hypoglycaemia | 1(4) | 0 | 0 | 1(1) |
| Renal failure | 2 (8) | 0 | 0 | 2 (3) |
| Hypovolemic shock | 0 | 1(3) | 0 | 1(1) |
| Total | 6 (24) | 6 (17) | 5 (36) | 17 (23) |

SAE: Serious adverse event, VL: visceral leishmaniasis
