# Supplementary material for: Use of Pentamidine As Secondary Prophylaxis to Prevent Visceral Leishmaniasis Relapse in HIV Infected Patients, the First Twelve Months of a Prospective Cohort Study
Source: PLoS Negl Trop Dis. 2015 Oct 2;9(10):e0004087. doi: 10.1371/journal.pntd.0004087 (PMC4591988; doi:10.1371/journal.pntd.0004087)
Supplement: S2 Table — (DOCX) [file pntd.0004087.s003.docx]

**Supplemental table 2: Adverse events in the 12 months follow up with use of MEDRA term and body system classification**

| **MEDRA Preferred terms classified in Body Systems** | | **Current Primary** | **Current Relapse** | **Past VL** | **Total** |
| --- | --- | --- | --- | --- | --- |
| **Blood and Lymphatic system disorders** | | **4** | **3** | **2** | **9** |
| Anemia | 3 | 2 | 2 | 7 |  |
| Lymphadenopathy | 1 | 0 | 0 | 1 |  |
| Thrombocytosis | 0 | 1 | 0 | 1 |  |
| **Eye Disorders** | | **1** | **5** | **3** | **9** |
| Eye allergy | 1 | 0 | 1 | 2 |  |
| Eye degenerative disorder | 0 | 0 | 1 | 1 |  |
| Iridocyclitis | 0 | 2 | 0 | 2 |  |
| Night blindness | 0 | 1 | 1 | 2 |  |
| Ocular hyperaemia | 0 | 1 | 0 | 1 |  |
| Refractory disorder | 0 | 1 | 0 | 1 |  |
| **Gastrointestinal disorders** | | **13** | **11** | **24** | **48** |
| Abdominal distension | 0 | 0 | 1 | 1 |  |
| Abdominal pain | 2 | 1 | 2 | 5 |  |
| Anal fistula | 0 | 0 | 1 | 1 |  |
| Ascitis | 0 | 0 | 1 | 1 |  |
| Diarrhea | 6 | 8 | 10 | 24 |  |
| Dyspepsia | 3 | 0 | 6 | 9 |  |
| Haemorrhoids | 0 | 1 | 1 | 2 |  |
| Nausea | 2 | 0 | 1 | 3 |  |
| Toothache | 0 | 1 | 0 | 1 |  |
| Upper gastrointestinal bleeding | 0 | 0 | 1 | 1 |  |
| **General and administration site conditions** | | **4** | **5** | **4** | **13** |
| Application site hyperemia | 1 | 2 | 0 | 3 |  |
| Asthenia | 0 | 0 | 1 | 1 |  |
| Injection site swelling | 1 | 0 | 0 | 1 |  |
| pyrexia | 2 | 2 | 3 | 7 |  |
| Weight loss | 0 | 1 | 0 | 1 |  |
| **Infections and infestations** | | **35** | **51** | **17** | **103** |
| Abscess neck | 1 | 0 | 0 | 1 |  |
| Acarodermatitis | 1 | 0 | 0 | 1 |  |
| Acute tonsillitis | 0 | 1 | 0 | 1 |  |
| Amoebiasis | 1 | 0 | 1 | 2 |  |
| Ascariasis | 0 | 1 | 1 | 2 |  |
| Body tinea | 1 | 0 | 0 | 1 |  |
| Carbuncle | 1 | 1 | 0 | 2 |  |
| Cellulitis | 1 | 0 | 1 | 2 |  |
| Cestode infection | 0 | 2 | 1 | 3 |  |
| Disseminated tuberculosis | 0 | 1 | 0 | 1 |  |
| Eye infection | 2 | 3 | 2 | 7 |  |
| Folliculitis | 0 | 1 | 0 | 1 |  |
| Fungal skin infection | 1 | 0 | 0 | 1 |  |
| Giardiasis | 1 | 6 | 1 | 8 |  |
| Helminthic infection | 0 | 2 | 1 | 3 |  |
| Herpes zoster | 1 | 1 | 0 | 2 |  |
| Impetigo | 1 | 1 | 0 | 2 |  |
| Influenza | 1 | 0 | 0 | 1 |  |
| Intertrigo candida | 0 | 1 | 0 | 1 |  |
| Isosporiasis | 1 | 0 | 0 | 1 |  |
| Lymphadenitis bacterial | 1 | 0 | 0 | 1 |  |
| Malaria | 4 | 5 | 1 | 10 |  |
| Meningitis | 1 | 0 | 0 | 1 |  |
| Nasopharingitis | 1 | 0 | 0 | 1 |  |
| Oesophageal candidiasis | 0 | 0 | 1 | 1 |  |
| Oral candidiasis | 0 | 0 | 1 | 1 |  |
| Otitis media | 2 | 0 | 0 | 2 |  |
| Peritonitis bacterial | 0 | 0 | 1 | 1 |  |
| *Plasmodium falciparum* | 1 | 1 | 0 | 2 |  |
| *Plasmodium vivax* | 2 | 2 | 1 | 5 |  |
| Pulmonary tuberculosis | 0 | 1 | 0 | 1 |  |
| Pulpitis dental | 1 | 0 | 0 | 1 |  |
| Skin infection | 0 | 1 | 0 | 1 |  |
| Strongyloidiasis | 0 | 3 | 0 | 3 |  |
| Tonsillitis | 1 | 0 | 0 | 1 |  |
| Typhoid fever | 1 | 0 | 0 | 1 |  |
| Urinary tract infection | 2 | 3 | 2 | 7 |  |
| Visceral leishmaniasis | 3 | 13 | 2 | 18 |  |
| Would infection | 1 | 1 | 0 | 2 |  |
| **Injury, poisoning and procedural complications** | | **1** | **7** | **2** | **10** |
| Animal bite | 0 | 2 | 0 | 2 |  |
| Excoriation | 0 | 1 | 1 | 2 |  |
| Muscle strain | 1 | 0 | 1 | 2 |  |
| Soft tissue injury | 0 | 3 | 0 | 3 |  |
| Wound | 0 | 1 | 0 | 1 |  |
| **Investigations** | | **2** | **3** | **1** | **6** |
| Blood albumin decrease | 0 | 0 | 1 | 1 |  |
| Blood alkaline phosphatase increase | 1 | 1 | 0 | 2 |  |
| Blood creatinine increase | 0 | 1 | 0 | 1 |  |
| Platelet count decrease | 0 | 1 | 0 | 1 |  |
| Transaminases increase | 1 | 0 | 0 | 1 |  |
| **Metabolism and nutritional disorders** | | **5** | **3** | **5** | **13** |
| Decreased appetite | 3 | 2 | 2 | 7 |  |
| Dehydration | 1 | 0 | 0 | 1 |  |
| Hyperglycemia | 0 | 0 | 1 | 1 |  |
| Hypoglycemia | 1 | 1 | 1 | 3 |  |
| Tetany | 0 | 0 | 1 | 1 |  |
| **Musculoskeletal and connective tissue disorders** | | **5** | **5** | **7** | **17** |
| Arthralgia | 2 | 3 | 6 | 11 |  |
| Back pain | 1 | 1 | 1 | 3 |  |
| Myalgia | 1 | 0 | 0 | 1 |  |
| Pain in the extremity | 1 | 1 | 0 | 2 |  |
| **Neoplasms benign, malignant and unspecified** | | **0** | **1** | **0** | **1** |
| Anogenital warts | 0 | 1 | 0 | 1 |  |
| **Nervous system disorder** | | **0** | **4** | **3** | **7** |
| Dizziness | 0 | 0 | 1 | 1 |  |
| Headache | 0 | 2 | 1 | 3 |  |
| Neuropathy peripheral | 0 | 1 | 1 | 2 |  |
| Polyneuropathy | 0 | 1 | 0 | 1 |  |
| **Psychiatric disorders** | | **0** | **0** | **1** | **1** |
| Somatoform disorder | 0 | 0 | 1 | 1 |  |
| **Renal and urinary disorders** | | **4** | **2** | **2** | **8** |
| Renal failure | 2 | 0 | 0 | 2 |  |
| Renal failure acute | 1 | 0 | 0 | 1 |  |
| Renal impairment | 0 | 0 | 1 | 1 |  |
| Cystitis | 1 | 0 | 0 | 1 |  |
| Urinary discharge | 0 | 2 | 1 | 3 |  |
| **Reproductive system and breast disorders** | | **0** | **0** | **2** | **2** |
| Gynecomastia | 0 | 0 | 1 | 1 |  |
| Scrotal swelling | 0 | 0 | 1 | 1 |  |
| **Respiratory, thoracic and mediastinal disorders** | | **18** | **20** | **18** | **56** |
| Cough | 0 | 2 | 2 | 4 |  |
| Epistaxis | 0 | 1 | 0 | 1 |  |
| Nasal congestion | 8 | 9 | 1 | 18 |  |
| Bronchitis | 0 | 0 | 1 | 1 |  |
| Bronchopneumonia | 0 | 0 | 1 | 1 |  |
| Atypical pneumonia | 1 | 1 | 2 | 4 |  |
| Pneumonia | 5 | 6 | 6 | 17 |  |
| Upper respiratory tract infection | 3 | 0 | 1 | 4 |  |
| Chest pain | 1 | 1 | 4 | 6 |  |
| **Skin and subcutaneous disorders** | | **4** | **7** | **2** | **13** |
| Acne | 0 | 1 | 0 | 1 |  |
| Dermatitis allergic | 1 | 0 | 1 | 2 |  |
| Dermatitis contact | 1 | 0 | 0 | 1 |  |
| Eczema | 1 | 0 | 0 | 1 |  |
| Pruritus | 1 | 1 | 1 | 3 |  |
| Rash | 0 | 2 | 0 | 2 |  |
| Skin hypopigmentation | 0 | 1 | 0 | 1 |  |
| Skin lesion | 0 | 1 | 0 | 1 |  |
| Skin reaction | 0 | 1 | 0 | 1 |  |
| **Vascular disorders** | | **3** | **5** | **4** | **12** |
| Hypotension | 3 | 4 | 4 | 11 |  |
| Hypovolemic shock | 0 | 1 | 0 | 1 |  |
| **Total** | | **99** | **132** | **97** | **328** |
