# Supplementary material for: Use of Pentamidine As Secondary Prophylaxis to Prevent Visceral Leishmaniasis Relapse in HIV Infected Patients, the First Twelve Months of a Prospective Cohort Study
Source: PLoS Negl Trop Dis. 2015 Oct 2;9(10):e0004087. doi: 10.1371/journal.pntd.0004087 (PMC4591988; doi:10.1371/journal.pntd.0004087)
Supplement: S3 Table — (DOCX) [file pntd.0004087.s004.docx]

**Supplemental table 3: Adherence to antiretroviral therapy and outcome (assessment done at each monthly visit of 12 months follow up)**

| Study (month 12) end point | No ART missed | <3 doses/month missed | 3 - 9 doses/month missed | >9 doses missed/month | Total |
| --- | --- | --- | --- | --- | --- |
| Lost to follow up | 34 | 0 | 0 | 2 | 36 |
| Discontinuation of pentamidine for safety | 7 | 1 | 0 | 0 | 8 |
| Death | 24 | 2 | 0 | 0 | 26 |
| Other reason for discontinuation of pentamidine (patient refusal) | 6 | 0 | 0 | 0 | 6 |
| Relapse | 92 | 8 | 0 | 0 | 100 |
| CD4<200 | 236 | 13 | 0 | 1 | 250 |
| CD4>200 | 363 | 21 | 9 | 1 | 394 |
| Total | 762 | 45 | 9 | 4 | 820 |

ART: antiretroviral therapy
